# Implementation of a novel video telehealth program for musculoskeletal disorder self-management in U.S. military veterans during the COVID-19 pandemic combining yoga therapy with education

**DOI:** 10.1186/s12998-026-00685-5

**Published:** 2026-11-09

**Authors:** Steven Huybrecht, Bridget Conboy, Zachary A. Cupler

**Affiliations:** 1 Integrative Health Service, San Francisco VA Health Care System, San Francisco, USA; 2 Physical Medicine and Rehabilitative Services, Butler VA Health Care System, Butler, USA; 3 Geriatric Research Education and Clinical Center (GRECC), VA Pittsburgh Healthcare System, Pittsburgh, PA USA; 4 VA Pacific Islands Health Care System, 91-1051 Franklin D. Roosevelt Avenue, Kapolei, HI 96707 USA

**Keywords:** Chronic pain, Musculoskeletal pain, Musculoskeletal disorders, Telehealth, COVID-19, Veterans, Chiropractic, Yoga, Veterans health administration

## Abstract

**Background:**

Musculoskeletal disorders (MSDs) are highly prevalent, costly, and burdensome. Over 40% of United States military veterans are diagnosed with chronic pain related to the spine. Various clinical practice guidelines recommend nonpharmacological treatments for the management of chronic MSDs including spinal pain. During the COVID-19 pandemic, face-to-face healthcare was diverted to telehealth platforms to minimize exposure of communicable disease. To address barriers to in-person treatment, veterans experiencing pain associated with MSDs at one Veterans Health Administration Medical Center were offered a novel evidence-based approach incorporating patient education and experiential movement through a telehealth modality.

**Methods:**

We describe a novel telehealth clinic for self-management of MSDs offered from February 2021 to September 2021. The clinic consisted of a one-hour class offered weekly for four weeks, and included didactic lecture content and movement therapy. The classes were offered by a chiropractor and a yoga instructor. We retrospectively evaluated implementation of the telehealth clinic, including program attendance. Attendance of three or more of the four sessions (75%) was selected as a pragmatic descriptive indicator of patient participation.

**Results:**

Seventeen veterans participated in three cohorts over six months. The mean age of participants was 54.8 years (SD: 14.6; range 34 to 80), and 12 (70.6%) were male. Seven participants (41.2%) attended at least three of the four sessions, including five (29.4%) who attended all four sessions. Participants represented a geographically diverse population, with varying distances from VA facilities and VA facilities with chiropractic services.

**Conclusions:**

We describe a clinical demonstration of a telehealth clinic for MSDs offering evidence-based content with experiential movement. This program was delivered through video telehealth and a subset of participants sustained attendance across most of the four-session series. The preliminary information reported could be used for development and expansion of this type of clinic for telehealth chronic MSD management. Future studies are needed to determine the impact of telehealth delivery of MSD care on outcomes, cost, and patient preference. Expanding telehealth delivery of MSD care may be particularly valuable for rural veterans, those lacking access to transportation, or patients who experience barriers to attending in-person appointments.

## Introduction

Globally, musculoskeletal disorders (MSDs) account for 21% of morbidity and affect more than 25% of the population [1]. Specifically, low back pain (LBP) is a highly prevalent, costly, and burdensome condition. In the United States, LBP costs exceed $100 billion USD annually [2] and is the leading cause of disability, activity limitation, and work absence [3]. Military veterans experience a substantial burden of pain. Nationally representative data from 2010 to 2014 found that severe pain was 40% more prevalent among veterans (9.1%) than nonveterans (6.4%) [4]. From 2002 to 2018, the absolute increase (95% CI) in low back pain prevalence among veterans was 10.3% points (9.5%-11.1%) [5].

The American College of Physicians [6], Department of Veterans Affairs, and the Department of Defense clinical practice guidelines for LBP [7] recommend a variety of non-pharmacological treatments for the management of LBP including patient education, structured exercise, manual therapies, and cognitive behavioral therapy within team-based care models. It is common practice for rehabilitation and manual therapy clinicians (e.g., chiropractors, massage therapists, physical therapists) to manage patients with MSDs with in-person treatment. Unfortunately, the onset of the COVID-19 pandemic resulted in a rapid disruption and reduction of face-to-face care delivery and swift telehealth uptake by nearly all clinical disciplines. This shift in care delivery required interventions that could be delivered remotely, including education, guided exercise, and self-management strategies that did not require direct physical contact between a clinician and a patient.

Prior to the COVID-19 pandemic, evidence regarding scalable telehealth MSD management options was limited and benefit was mixed. A 2017 systematic review of real-time telerehabilitation for MSDs found outcomes comparable to standard face-to-face practice for improvements in function and pain [8]. Conversely, another 2017 systematic review focused on low back pain management found that telehealth interventions alone were not more effective than minimal interventions for reducing pain and disability in chronic LBP [9]. Subsequent reviews also evaluated telehealth applications in musculoskeletal and rehabilitation care with similar equivocal findings [10, 11]. Within veteran populations, remote and hybrid approaches have been evaluated. A pilot study at one Veterans Health Administration (VA) Health Care System reported on enrollment and participation outcomes for a home-based telephone-supported physical activity program for older veterans with chronic LBP [12]. A 2024 clinical trial of a hybrid LBP care pathway incorporating telehealth-delivered physical activity and psychologically informed care reported variable utilization of telehealth and face-to-face clinical services among participants at five VA primary care clinics [13].

Given the disruption of face-to-face outpatient services because of the COVID-19 pandemic, San Francisco VA Health Care System implemented a novel telehealth program for self-management of chronic MSDs. The telehealth *Made to Move* group class (*M2M*) combined patient education lectures and experiential movement (i.e., yoga). Thus, this report aimed to describe the implementation and patient participation from a clinical demonstration project involving a novel telehealth program designed to deliver evidence-based education and self-management strategies for chronic MSDs within the ambulatory care setting of one VA Health Care System.

## Methods

The *M2M* program was implemented in 2021 as a clinical service in response to the disruption of face-to-face MSD care during the COVID-19 pandemic. This report retrospectively describes this clinical demonstration project. The *VA Electronic Determination Tool* (completed August 24, 2023) concluded that this project did not meet the regulatory definition of research and therefore did not require Institutional Review Board oversight. As such, this was treated as operational quality improvement and reporting was informed by the Revised Standards for Quality Improvement Reporting Excellence (SQUIRE 2.0) [14]. This manuscript was reviewed and approved by a VA Privacy Officer.

### Practice description

The *M2M* MSD group telehealth class was offered in a cohort model from February 2021 to September 2021 by San Francisco VA Health Care System. The goal of the telehealth *M2M* class was to promote veteran self-efficacy and empower self-management of chronic MSDs.

### Clinic development and design

To minimize the chance of spreading a communicable disease during the COVID-19 pandemic, we created and implemented a novel telehealth MSD group clinic as an additional option due to disrupted face-to-face clinical care to meet the needs of veterans at one VA health care system. During development of *M2M* in early 2021, we reviewed existing in-person and telehealth programming available through Integrative Health and Whole Health within the San Francisco VA Health Care System and did not identify similar group offerings for MSDs.

Education was paired with yoga-based movement to provide both cognitive and experiential components within the telehealth program. Informed by Whole Health principles [15], the *M2M* group class emphasized patient education, reassurance, empowerment, and development of skills for self-management of pain related to MSDs. Whole Health principles informed the *M2M* program’s emphasis on active patient participation and self-management, while clinical guidelines and best-practice recommendations informed inclusion of pain neuroscience education (PNE), movement and exercise, and other nonpharmacologic self-care strategies [6, 16]. Yoga, an active, nonpharmacologic self-management approach, was selected because it could be readily adapted for remote group delivery and an existing clinical staff member available to support the clinic had formal yoga training.

An experienced staff licensed clinical social worker (BC) with training as a yoga instructor (Registered Yoga Teacher, 200 h) and staff chiropractor (SH) collaboratively developed a series of four, one-hour video telehealth classes tailored to veterans with chronic MSDs. Session education content incorporated concepts of PNE [17–19], active care – yoga [20], and self-care strategies – positional modifications, cryo- or heat therapy, self-massage.

### Setting

San Francisco VA Medical Center is a level 1a facility within the San Francisco VA Health Care System that served nearly 110,000 veterans in FY2024 across the Medical Center and nine Community-Based Outpatient Clinics (CBOCs). The Health Care System’s outpatient services provide primary care, mental health, and specialty care services with over 940,000 outpatient visits in FY2024 [21]. The chiropractic service was aligned under the Integrative Health (IH) service line. Prior to the COVID-19 pandemic and development of *M2M*, yoga instruction was available virtually through IH and Whole Health group programming.

### M2M Virtual MSD health group

**Fig. 1:**
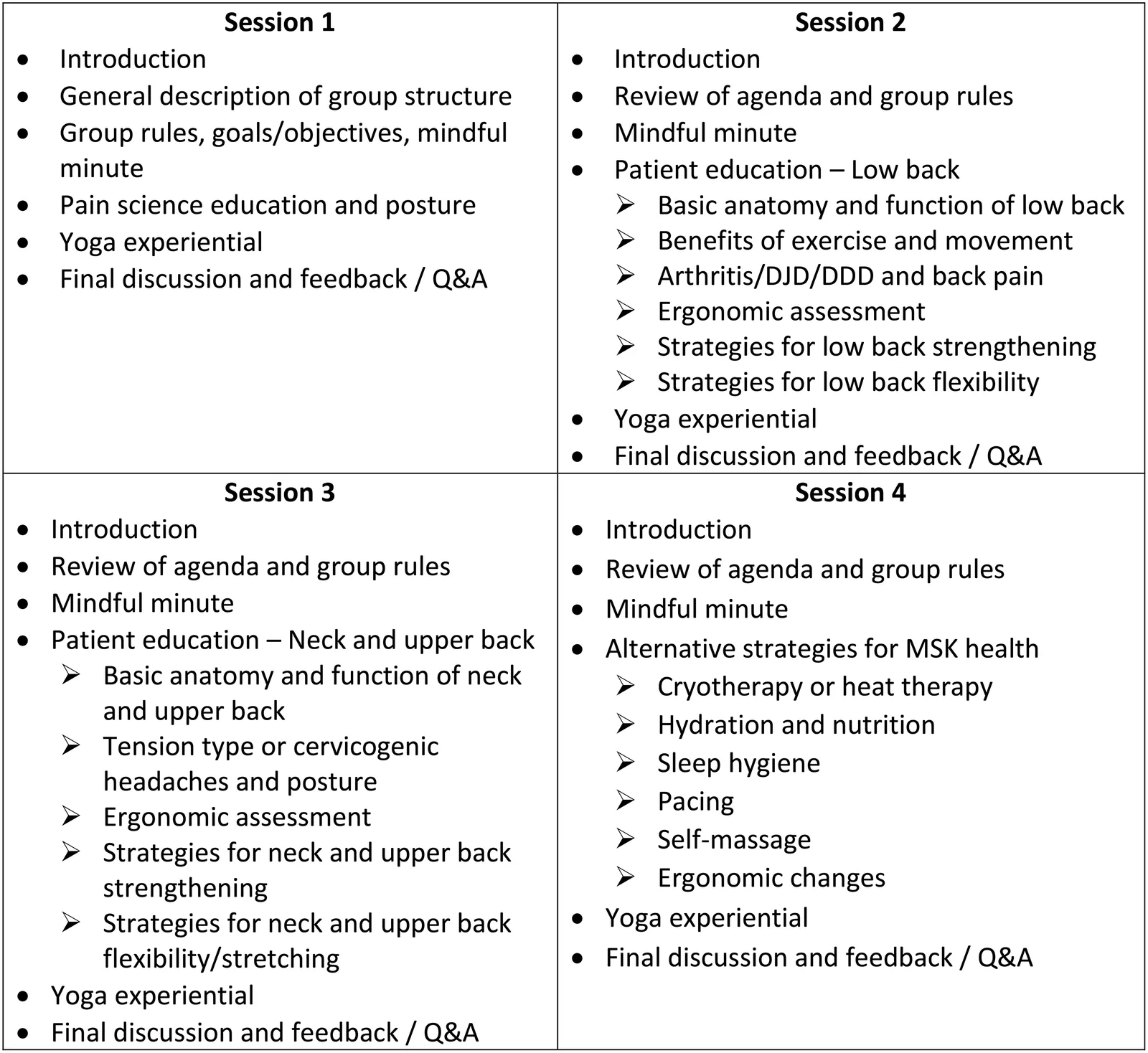
*Made to Move* group session outlines

The *M2M* series of classes was offered on a weekly basis for four weeks using the secure videoconferencing platform, Webex by Cisco (San Jose, California). The *M2M* weekly class was a one-hour class split with didactic lecture content and movement therapy (Fig. 1). The chiropractor provided lecture style education for 20–30 min with an emphasis on PNE and self-efficacy for chronic musculoskeletal pain self-management. The yoga instructor led the second half of each class for 20–25 min of yoga. Yoga poses taught and practiced during the four sessions were modified as necessary and included savasana, bent knee savasana, and various asana positions focused on spinal health.

The *M2M* instructors sent eligible patients a unique link via VA Video Connect prior to the class each week. Patients attended from their home with the use of a tablet, computer, or smart phone and required enough space for one person to lay down.

### Participants

Patients were referred to the *M2M* group via Whole Health Consult integrated within the electronic health record, which was used to refer patients to a variety of Whole Health services and group offerings. Referring providers could select *M2M* as the specific program for which the patient was being referred, and appropriate candidates were accepted. Marketing efforts included a flyer posted in high traffic areas throughout the Medical Center and an e-mail sent to IH clinical team members. Patients were informed of the *M2M* offering during the limited in-person appointments offered by *M2M*’s instructors and IH clinical providers.

Potential patients were identified from referrals and contacted by phone (BC) to review the program structure, content, expectations, schedule, and technological requirements. Those interested in participating were sent an introductory e-mail with information regarding class dates, times, and recommendations for technology preparation before the first session. Prior yoga experience was not required.

### Eligibility criteria

The *M2M* group was developed primarily for patients experiencing chronic MSD pain complaints, reflecting the predominant clinical population and the program’s emphasis on self-management. Acute MSDs were not specifically excluded. Eligible patients were those who were interested in participating, available during the scheduled class times, able to access the telehealth platform, and cleared by their primary care team to exercise independently.

### Outcomes

Attendance at three or more of the four sessions was selected as a pragmatic indicator of patient participation. This threshold was chosen because attendance of less than three sessions resulted in missing 50% or more of the educational and experiential content. This threshold was not prospectively defined as a research endpoint or measure of clinical effectiveness.

### Data collection and analysis

Patients were identified within the San Francisco VA Health Care System who attended at least one telehealth class of *M2M* between February 1, 2021 and September 1, 2021. This time frame encompassed the duration in which the *M2M* clinic was offered; after September 2021, increased demand for face-to-face clinical services resulted in reprioritization of staffing resources to in-person care.

We performed a manual chart review and extracted patient demographics and attendance for all patients identified by clinic visit in the electronic health record. Demographics included age, sex, race, county of residence, service connection rating percentage, any pain associated service connection rating diagnosis, distance to nearest VA facility, and distance to nearest VA facility with a staff chiropractor.

This is a clinical demonstration project describing a novel telehealth MSD group clinic. We did not perform hypothesis-testing or inferential statistics. As such, we did not perform a priori power analysis for a sample size estimate. As we did not power the study for evaluating clinical effectiveness, we did not consider collecting clinical outcomes (i.e., pain, function, and quality of life).

## Results

Three, four-session *M2M* cohorts were offered between February and September 2021. A total of 17 patients attended at least one group class (Table 1). The mean age of participants was 54.8 years (SD: 14.6). Most participants were male (*n* = 12, 70.6%), while race was most commonly reported as white (*n* = 6, 35.3%) or unspecified (*n* = 5, 29.4%). The most common primary MSD diagnosis was spine-related (*n* = 7) followed by neurological (*n* = 2). Most patients’ listed residence zip code was within 21 miles of the nearest VA Medical Center or CBOC (*n* = 11, 64.7%) and a majority were within 21 miles of the nearest VA Medical Center or CBOC with a staff chiropractor (*n* = 9, 52.9%).

**Table 1:** Demographic characteristics of *Made to Move* group patients. (*n* = 17)

Patient Characteristics	
Age, years, Mean ± SD [range]	54.8 ± 14.6 [34–80]
Sex, n (%)	
Male	12 (70.6%)
Female	4 (23.5%)
Declined to Answer	1 (5.9%)
Race, n (%)	
Caucasian	6 (35.3%)
Asian	3 (17.6%)
African American	2 (11.8%)
American Indian	1 (5.9%)
Unspecified	5 (29.4%)
Service Connection Rating, %, mean	75.3%
Pain-Related Service-Connected Conditions, n (%)*	
Spinal conditions	8 (47.1%)
Extremity conditions	8 (47.1%)
Nerve conditions	6 (35.3%)
Headache	3 (17.6%)
Somatization disorder	1 (5.9%)
Distance to nearest VA facility, n (%)	
< 21 miles	11 (64.7%)
21–40 miles	4 (23.5%)
41–60 miles	0 (0%)
> 61 miles	1 (5.9%)
Unspecified	1 (5.9%)
Distance to nearest VA with a chiropractor, n (%)	
< 21 miles	9 (52.9%)
21–40 miles	4 (23.5%)
41–60 miles	1 (5.9%)
> 61 miles	2 (11.8%)
Unspecified	1 (5.9%)

*Multiple conditions possible for each patient

Of the 17 patients, seven (41.2%) attended three or more of the four *M2M* group sessions, including two patients (11.8%) who attended three sessions and five patients (29.4%) who attended all sessions (Table 2). The remaining ten patients attended either one session (*n* = 7; 41.2%) or two sessions (*n* = 3; 17.6%).

**Table 2:** *Made to Move* telehealth group attendance

Number of Made to Move telehealth sessions attended	*n* (%)
1	7 (41.2)
2	3 (17.6)
3	2 (11.8)
4	5 (29.4)

## Discussion

This clinical demonstration project describes delivery of a novel telehealth spine health group class, *M2M*, among patients at a single VA Health Care System. During the COVID-19 pandemic, patients were offered *M2M* as a telehealth alternative to in-person MSD care, as there were no comparable in-person or telehealth group programs combining MSD education with experiential movement at the time. Seven of the 17 participants (41.2%) attended at least three of the four sessions, representing sustained participation among a subset of enrolled veterans. Novel approaches to face-to-face care for chronic MSD management are of particular benefit to veterans, a group that experiences chronic MSDs [22, 23] at higher rates than civilians. Patients who receive care at VA may also experience additional barriers to attending appointments for in-person care (i.e., psychiatric co-morbidities including post-traumatic stress disorder [24, 25], anxiety [26], depression [27], limited transportation [28–30], childcare needs [31], and distance to physical hospital [32]). To our knowledge, this is the first implementation combining chronic MSD patient education from a non-pharmacological rehabilitation provider (e.g., chiropractor) with experiential learning by a registered yoga teacher who also operated as a clinical social worker in the medical center. With future clinical effectiveness evaluation, this telehealth-based option may be scalable and optimized for delivery within a large health care system dependent on the available clinical workforce.

Multiple viable options exist for the experiential movement instructor who could be trained in the movement discipline and have a clinical background as a physical, occupational, or recreational therapist, therapy assistant, or nurse. The planning and programmatic development stages of the *M2M* clinical offering buildout (early 2021) yielded limited comparable telehealth groups in integrated care settings. A cursory evaluation of the literature during the development of this manuscript (late 2023) identified mixed findings regarding the effectiveness of telehealth for management of MSDs, a wide range of interventions described, and relatively few examples incorporating experiential learning. In the absence of other examples during the design phase, we devised the *M2M* educational component in a manner that is well suited for delivery by rehabilitation and manual therapy clinicians. The experiential yoga component of our program was delivered by a social worker with applicable yoga training who operated in both a clinical role and as a yoga teacher in the medical center. Many iterations of experiential movement interventions could be considered in future adaptations of telehealth care delivery for MSDs. For example, tai chi, qigong, Pilates, or other structured movement approaches could potentially be substituted for yoga depending on available staff expertise, local resources, and patient preferences. Future work should evaluate whether different movement-based components influence participation and clinical outcomes, and if patients have preferences for specific movement-based systems in this model of care delivery.

Historically, ‘back schools’ have combined education and exercise to support the management of LBP. Straube et al. [33] concluded that nearly a half-century after back schools were first trialed, no unequivocal evidence of benefit has emerged. Chou and Huffman [34] found that several non-pharmacologic approaches, including cognitive-behavioral therapy, exercise, spinal manipulation, and interdisciplinary rehabilitation were all moderately effective for chronic LBP. These established approaches provide clinical context for the development of *M2M*; however, the present evaluation was not designed to compare *M2M* with these interventions or assess clinical effectiveness. The *M2M* group differed from traditional back schools by incorporating interdisciplinary delivery, PNE for a variety of MSDs, and an experiential mind-body intervention through yoga. Future research should include adequately powered prospective designs to evaluate patient-reported outcomes such as quality of life, function, and patient-specific functional measures; short- and long-term clinical outcomes; experiences and preferences; downstream healthcare utilization, including opioid use, imaging, and surgical referrals; and costs associated with MSD management.

Patient decisions regarding accessing health care services are complex and often based on a variety of factors, including distance or travel time to the facility [35] and wait time for an appointment [36]. Access to and familiarity with technology might inhibit engagement, though resources such as VA’s Digital Divide consult [37] may be available to aid in participation; this ensures access to digital technology for enrolled patients – something that may not translate to other health care systems. As some technologies become easier to use and less expensive, accessibility improves as the technology migrates from hospitals and institutional settings and into patients’ homes [38].

Telehealth programs such as *M2M* may represent one approach for addressing access barriers to non-pharmacologic pain management, particularly for veterans who live farther from facilities offering these services. Future evaluations should specifically assess whether programs such as *M2M* improve access to care. Economic, racial, ethnic, and geographic disparities in access to high-quality non-pharmacologic pain management options remain highly prevalent [39, 40]. Telehealth may reduce transportation-related barriers, which are often encountered by patients of low socioeconomic status or in geographic areas with a care desert.

Although chiropractors are commonly associated with spinal manipulation, patient education is also within the scope of chiropractic practice. For *M2M*, the existing staff chiropractor delivered the educational component through telehealth without providing manual therapies. This *M2M* evaluation did not assess whether telehealth-based education delivered by a chiropractor affects clinical outcomes, healthcare utilization, or costs. Prior studies of chiropractic care pathways have reported differences in downstream healthcare utilization, including imaging, surgery, injections, opioid use, and associated costs [41–44]; however, those findings should not be extrapolated to the *M2M* program. Future research could evaluate whether telehealth-based MSD education delivered by chiropractors or other rehabilitation clinicians influences clinical outcomes, healthcare utilization, and costs.

### Limitations

This was a clinical demonstration project and, therefore, not adequately powered to provide any measure of statistical significance regarding clinical effectiveness. Cautious interpretation of these findings is necessary given the demonstration project design, small sample size (*n* = 17) and limited number of cohorts. We did not utilize pre- and post-outcome measures to gather quantitative or qualitative data for assessment of benefit, or lack thereof, as our focus was on program implementation and attendance. Second, this intervention was delivered during a unique time period (i.e., COVID-19) with the available resources and staffing at the time, which may not be reproducible with return to in-person care offerings. Third, future implementation of *M2M* may be limited by competing clinical demands on staff who are responsible for both telehealth and face-to-face care. Allocating protected time for group preparation and delivery may be challenging in settings with limited staffing or high demand for in-person services, and should be considered when adapting this model to other clinical environments. Fourth, practical limitations through this experience, often related to technology (e.g., participants being able to access the link, unmuting/muting, etc.), may have led some participants to discontinue their attendance. To minimize disengagement with telehealth modalities, we attempted to address this through test runs or one-on-one coaching. Fifth, we did not engage patients to assist with the intervention development stage nor did we seek input through focus groups or individual interviews during or after the intervention to gather patient perspective. Sixth, we did not establish a formal consultative process in the electronic health record that would allow determination of those potential patients who were referred to the *M2M* but did not participate, limiting our ability to determine a nonparticipation rate. Future work may consider qualitative assessments of barriers to and facilitators of participating in telehealth interventions for MSDs.

### Practical lessons learned

There are unique logistical challenges when offering telehealth care delivery for MSDs. We recommend incorporating five minutes of space preparation prior to the start of group content to minimize interruptions during the transition to the yoga experiential. Staff support to the lead instructor during class offering should be considered (i.e., ensuring all participants can access the hyperlink and participants remain muted during class) so as not to disrupt other attendees. Group materials should be further refined and distributed by email or through the group chat to support participants in practicing the self-care strategies introduced during the program. For future use of this type of clinic offering, we recommend patients complete pre- and post-cohort surveys including outcome measures (i.e., patient-reported outcome measures, functional activities, self-efficacy) and feedback assessments to promote continuous process improvement. Lastly, to support engagement we would outline a formal plan to contact patients as part of a more comprehensive follow-up for absent participants. The preliminary findings reported here could be used for development and expansion of this type of group clinic for chronic pain management via telehealth.

## Conclusions

We delivered *M2M*, a telehealth MSD group clinic, during the COVID-19 pandemic. Seven of the 17 participants (41.2%) attended three or more of the four sessions, demonstrating sustained participation among a subset of veterans. These findings provide preliminary information regarding engagement with a telehealth MSD self-management program and may help inform the design and evaluation of future telehealth interventions for MSDs. Future work to compare face-to-face care and telehealth interventions for chronic MSDs is needed to determine clinical effectiveness.

## Data Availability

The datasets used and/or analyzed during the current study are available from the corresponding author on reasonable request.

## Abbreviations

CBOC: Community-based outpatient clinic
IH: Integrative health
LCSW: Licensed Clinical Social Worker
M2M: Made to Move telehealth group class
MSD: Musculoskeletal disorder
PNE: Pain neuroscience education
VA: Veterans Health Administration
